# Something about Leprosy

**Published:** 1888-12-08

**Authors:** 


### The Hospital

A Weekly Institutional Journal of

**Science, Medicine, Nursing, and Philanthropy.**

*For Hospitals, Asylums, Medical Practitioners, Students, Nurses, Families, Parishes, Congregations, and the Charitable Public.*

Vol. V. No. 115.] [December 8, 1888.

### Something about Leprosy.

Our readers may have noticed a correspondence "going the round of the press" regarding the danger of leprosy being conveyed by the sick to the healthy. The Rev. H. P. Wright, rector of Greatham, wishes to "make known to the world at large the important and highly-interesting fact that at last it has been shown, by a terrible experiment on a criminal condemned to death, that leprosy may be conveyed by inoculation: a fact that in itself goes far to prove that the malady is contagious." It appears that one Keanu, a Hawaiian, in confinement at the Oahu Jail, in Honolulu, was inoculated with leprosy in November, 1885, and in September of the present year the man was certified to be a leper by the President of the Board of Health and the Government Physician, Honolulu. We are glad, however, to see that Dr. Robert Liveing, a high authority on leprosy, has written to the *Times* in a strain likely to mitigate any scare which the somewhat sensational notes of the Rev. Mr. Wright might cause. Dr. Liveing remarks, "I hope Archdeacon Wright will excuse me for saying that it does not go far to prove anything of the kind" (meaning the contagiousness of leprosy). "There is at present no evidence to show that leprosy is contagious in the ordinary sense of the word." With this we quite agree. But it would be well if the term "contagion" were not used in the loose manner so often the case. *Contagion* should be understood as implying the communication of disease by actual contact, while the term *infection* should be significant of the spread of disease without actual contact with the sick. But so far as is known, and according to the best authorities, leprosy is neither contagious nor infectious.

It has long been known that leprosy may be communicated from the sick to the healthy; but for this communication it is necessary that the discharge from a leprous sore should come into contact with a wound, or an abrasion of the skin, of a healthy person. That this not unfrequently happens in an accidental manner there is strong reason to believe. Damsch proved that leprosy may be communicated to animals by inoculation. It is stated by this experimentalist, that when portions of leprous tissue containing cells or bacilli are inserted into the bodies of cats or rabbits, morbid processes occur analogous to leprosy in man. Those who have seen most of leprosy are opposed to the view of the disease being infectious, distinctly stating that it is not communicable through the atmosphere in the manner in which small-pox, scarlet fever, and other diseases of the class are communicable. For example, the late Dr. Norman Chevers was "unacquainted with any fact in support." Dr. Rustomjee Khory, of Bombay, observes, "it is not contagious, the attendants on the sick do not contract it." Dr. Vandyke Carter, who so thoroughly investigated Eastern leprosy, is of opinion that it is only contagious "by the direct or indirect conveyance of the disease outside the body from person to person." Sir William Moore observes: "I do not hold that leprosy is communicated by infection, *i.e.*, through the medium of the atmosphere, as certain zymotic diseases are spread . . . the propagation of leprosy must take place by contagion through the abraded skin." This explains the reason why so many escape, although in constant communication with lepers, whereas accidental contact of leprosy poison with wounded skin explains many cases which do occur. A very slight contact is sufficient. It is on record that a boy pricked himself with a needle a leper had previously used, and became a leper. The fact that leprosy commences most frequently on exposed parts, especially the hands and feet of Easterns, who do not usually wear shoes and stockings, encourages the idea of communication from outside.

As with the questions of contagion and infection, the hereditary nature of leprosy has been the subject of much difference of opinion. The fact that lepers frequently have healthy children has been much dwelt on, as opposed to heredity. Those who have studied leprosy as it presents itself in the East, noted the number of leper families, and seen the number of leper children, are generally convinced of the heredity of the malady, even although children are not born lepers. On the other hand, the fact of persons developing leprosy who do *not* belong to known leper families cannot be ignored, and must be referred to infection or contagion. The balance of evidence on the subject is against infection, and strongly in favour of contagion, meaning by contagion the direct or indirect conveyance of *materies morbi* from the sores of the leper to the abraded skin of the healthy.

Leprosy was at one period a common disease in Great Britain. Old records show that leper houses were formerly numerous in France, Germany, and England. It is now met with in northern Europe generally, and also in Spain. It may, however, be doubted if leprosy, as observed in cold climates, is the same inveterate disease which prevails in the East. At the present time it is perhaps more destructive and prevalent in India than elsewhere. The Chinese consider a visit to the north a cure for leprosy, and it is certain that the intensity of the malady now corresponds with the belt of maximum heat throughout the Eastern Hemisphere. What principally concerns us is the possibility of leprosy being again introduced into and spreading in Great Britain in the manner feared by the Rev. Mr. Wright. Now we believe there is no such danger. The decline of leprosy in England, as also the decline of malarious fevers once prevalent in this country, was concomitant with the advance of civilisation generally, implying drainage, better water, better ventilation, better food, and improved sanitation and personal hygiene. For leprosy, like various other diseases, flourishes best under unsanitary conditions. Whatever induces *cachexia*, or a state of constitution below *par*, as defective diet, filth, damp, scurvy, impure water, exposure, will predispose to leprosy. Improved sanitation has enabled us of late years to bid defiance to cholera, and improved sanitation in the broadest sense of the term is our principal safeguard against leprosy. There is, however, unfortunately, one serious flaw in our armour. The connexion between specific disease and leprosy has long been a vexed question. Many of the signs and symptoms of the one disease are equally the characteristics of the other. Lindwörm, and, if we recollect right, Simon, long since asserted the connexion of the two maladies, regarding leprosy as the offspring of specific disease. Others, while denying a more intimate connexion, have admitted that the specific taint will prepare the system for leprosy. Sir William Moore has long held that leprosy is an inherited form of this malady.

There is considerable misapprehension as to what leprosy really is, most people believing the leper becomes "as white as snow." This is incorrect. "White skin disease," or *leucoderma*, is quite a different thing from modern leprosy. Again, leprosy has been confounded with the skin affection known as *lepra*, and also with *Elephantiasis* or "tropical big leg." Leprosy first manifests itself by one or more of three main symptoms, viz.: diffused red blotches on the skin, tubercles in the skin, and defective sensibility of the skin. These conditions may endure for an indefinite period, but eventually blisters and ulcers develop. Frequently the fingers and toes are first attacked, and eventually ulcerate away, a progress of leprosy often spoken of as "lepra mutilans." Before the period of ulceration the constitution is more or less undermined. The mucous membranes also become affected, and the disease attacks the throat and nose, exactly as does tertiary specific disease. The natives of India, according to Dr. Rustomjee Khory, "look upon leprosy as a punishment from God, and regard it with awe and superstition." In the earlier stages of the malady, in most parts of India, the leper, so long as he (or she) can obtain a living, is not an outcast from society; his fellows live in the same house with him, partake of the same food, and even allow him to marry. If the malady is chronic, and does not entail great suffering, many Indian lepers would rather endure their disease, which enables them to obtain a living as mendicants, than be free from it on the condition of working. Thus, many lepers who take advantage of leper asylums during the wet weather, prefer their liberty and the precarious existence of begging during the remainder of the year. When the disease progresses, and they suffer from pain and exhaustion, they become morose, inclined to brood in solitude, drink spirits, eat opium, and smoke *gunjah*; in fact, appear to endeavour to shun a sense of their unfortunate condition. Formerly, before it was put a stop to by the British Government, *sumajh*, or burial of lepers alive, was a common practice, and always with the consent of the leper himself, who, declaring to his relatives he was tired of life, would ask them to perform *sumajh*. Recently the segregation of all lepers in India has been much agitated. But, howeverdesirable, the measure is impracticable. Both the expense it would entail, and the fact that force only would confine the Indian lepers within prescribed bounds, forbid the procedure. It is also questionable if the benefits which those advocating wholesale segregation expect would result. If leprosy is, as there is every reason to believe, hereditary, it may be transmitted by persons who are not themselves developed lepers: or, in other words, it may not appear in one generation, although showing itself in the next. Against this, segregation would be no protection. Moreover, the universal segregation of lepers in India would be opposed to the customs, habits, and ideas of the people generally. The Government of India, acting on the advice of competent experts, have wisely declined any attempt at general segregation although fostering leper asylums and encouraging the people to resort to such havens. In India the diminution of leprosy is rather to be sought for by the progress of sanitation generally, in which is included the cheapening of salt, the supply of pure water, and the prevention of scarcity of food.

